# The potential of ALFA-tag and tyramide-based fluorescence signal amplification to expand the CRISPR-based DNA imaging toolkit

**DOI:** 10.1093/jxb/erae341

**Published:** 2024-08-06

**Authors:** Bhanu Prakash Potlapalli, Jörg Fuchs, Twan Rutten, Armin Meister, Andreas Houben

**Affiliations:** Leibniz Institute of Plant Genetics and Crop Plant Research (IPK) Gatersleben, 06466 Seeland, Germany; Leibniz Institute of Plant Genetics and Crop Plant Research (IPK) Gatersleben, 06466 Seeland, Germany; Leibniz Institute of Plant Genetics and Crop Plant Research (IPK) Gatersleben, 06466 Seeland, Germany; Leibniz Institute of Plant Genetics and Crop Plant Research (IPK) Gatersleben, 06466 Seeland, Germany; Leibniz Institute of Plant Genetics and Crop Plant Research (IPK) Gatersleben, 06466 Seeland, Germany; Cardiff University, UK

**Keywords:** ALFA-tag, chromosomes, CRISPR–FISH, dCas9, live cell imaging, tyramide system

## Abstract

Understanding the spatial organization of genomes within chromatin is crucial for deciphering gene regulation. A recently developed CRISPR–dCas9-based genome labeling tool, known as CRISPR–FISH, allows efficient labeling of repetitive sequences. Unlike standard fluorescence *in situ* hybridization (FISH), CRISPR–FISH eliminates the need for global DNA denaturation, allowing for superior preservation of chromatin structure. Here, we report on further development of the CRISPR–FISH method, which has been enhanced for increased efficiency through the engineering of a recombinant dCas9 protein containing an ALFA-tag. Using an ALFA-tagged dCas9 protein assembled with an Arabidopsis centromere-specific guide RNA, we demonstrate target-specific labeling with a fluorescence-labeled NbALFA nanobody. The dCas9 protein possessing multiple copies of the ALFA-tag, in combination with a minibody and fluorescence-labeled anti-rabbit secondary antibody, resulted in enhanced target-specific signals. The dCas9–ALFA-tag system was also instrumental in live cell imaging of telomeres in *Nicotiana benthamiana*. This method will further expand the CRISPR imaging toolkit, facilitating a better understanding of genome organization. Furthermore, we report the successful integration of the highly sensitive tyramide signal amplification method with CRISPR–FISH, demonstrating effective labeling of Arabidopsis centromeres.

## Teaser

Application of ALFA-tag and tyramide signal amplification provides potential strategies for enhancing CRISPR–FISH signals, thereby expanding the CRISPR-imaging toolbox for understanding chromosome biology.

## Introduction

Analysing the three-dimensional organization of genomes using microscopy techniques aids in understanding how the spatial arrangement of chromatin influences gene regulation and activity. Standard fluorescence *in situ* hybridization (FISH) is a powerful technique for mapping DNA sequences on fixed samples, commonly used in both research and diagnostic settings. However, it has a notable drawback: the necessity of global denaturation of double-stranded DNA to facilitate probe binding. This denaturation process often involves harsh treatments such as heat and formamide, which could potentially disrupt native biological structures and the chromatin organization (Mongelard *et al.*, 1999; Kozubek *et al.*, 2000; Solovei *et al.*, 2002; Markaki *et al.*, 2012; Boettiger *et al.*, 2016). Additionally, the hybridization and post-hybridization wash steps inherent to FISH protocols extend the time required to obtain results. To overcome these disadvantages, recent advancements in this field have led to development of DNA visualization methods that utilize CRISPR–dCas9-based labeling techniques tailored for animal and plant genomes (reviewed by Khosravi *et al.*, 2020a). One such innovative method is CRISPR–FISH (initially named ‘RGEN-ISL’; Ishii *et al.*, 2019), a rapid and straightforward method that utilizes the catalytically inactive Cas9 protein of *Streptococcus pyogenes* (dCas9) with a two-part guide RNA (gRNA), comprising a fluorophore-coupled trans-activating CRISPR RNA (tracrRNA) and target-specific CRISPR RNA (crRNA), to label repetitive DNA sequences in fixed samples (Ishii *et al.*, 2019; Potlapalli *et al.*, 2020). Notably, unlike standard FISH, CRISPR–FISH does not require a global denaturation of DNA and is, therefore, less damaging to the chromatin structure. Moreover, repetitive DNA sequences become detectable within a few seconds after applying the dCas9–RNA complex, demonstrating the rapid labeling capability of the CRISPR–FISH (Ishii *et al.*, 2019). This method is compatible with various fixation conditions, provides experimental flexibility across a temperature range from 4 °C to 37 °C, and can be combined with immunostaining and 5-ethynyl-2ʹ-deoxyuridine assays (Ishii *et al.*, 2019; Němečková *et al.*, 2019; Potlapalli *et al.*, 2020). Moreover, this method can label repetitive sequences on fixed plant tissue sections (Nagaki and Yamaji, 2020).

However, existing CRISPR–FISH methods have been limited to labeling repetitive sequences, presenting a challenge for marking low- or single-copy sequences. The difficulty arises from the limited number of fluorophores per ribonucleoprotein (RNP) complex. This challenge can be addressed by using multiple gRNAs targeting non-overlapping target regions for CRISPR–FISH. However, such an approach increases costs and the potential for off-target effects due to numerous gRNAs (Qin *et al.*, 2017). To address whether an increased number of reporter fluorophores per RNP complex could be used to boost the signal intensity, we employed multiple copies of the 15 amino acid-long ALFA-tag and corresponding nanobody-based fluorescing binders for amplification of CRISPR–FISH signals.

When combined with a protein of interest, the rationally designed ALFA-tag epitope can be specifically detected using a fluorescence-labeled NbALFA nanobody (Götzke *et al.*, 2019). Recently, studies in diverse species like *Drosophila melanogaster* (Xu *et al.*, 2022), yeast (Akhuli *et al.*, 2022), *Escherichia coli* (Westlund *et al.*, 2023), *Caenorhabditis elegans* (Igreja *et al.*, 2022), and HeLa cells (Jedlitzke and Mootz, 2022) have successfully employed the ALFA-tag for imaging analysis. Here, we present an innovative CRISPR–FISH labeling advancement by combining CRISPR–FISH-based sequence detection with ALFA-tag-aided fluorescence signal generation. Furthermore, we demonstrate live imaging of telomeres in transiently transformed *Nicotiana benthamiana* using the dCas9–ALFA-tag *in vivo*. Additionally, we also tested the combination of the tyramide-assisted amplification (TSA) system with CRISPR–FISH. TSA relies on the enzymatic deposition of reporter-conjugated tyramide near target nucleic acid sequences or protein on fixed samples, mediated by horseradish peroxidase (HRP) (Adams, 1992). The key to TSA is the multiple deposition of the reactive reporter-conjugated tyramide, resulting in an increased sensitivity of up to 1000-fold (Adams, 1992; Raap *et al.*, 1995). These novel techniques were tested for their potential to expand the CRISPR imaging toolbox in the field of chromosome biology.

## Materials and methods

### Materials

Seeds of *Arabidopsis*  *thaliana* were sown in soil and germinated under short-day conditions (16 h dark/8 h light, 18–20 °C) and then transferred to long-day conditions (16 h light/8 h dark, 18–20 °C) before bolting. Seeds of *Nicotiana benthamiana* were sown in soil and germinated under long-day conditions (16 h light/8 h dark) at 22 °C and grown for 2–4 weeks in a greenhouse. Maize (*Zea mays*) plants were grown in a greenhouse in pots, and leaf material was collected from 3-week-old plants. For chromosome preparations, maize (*Zea mays*) and broad bean (*Vicia faba*) seeds were germinated on wet filter paper in a Petri dish at 26 °C, and root tips were collected from 3 to 5 day-old seedlings. A chromosome suspension of the house mouse (*Mus musculus forma domestica*) strain C57Bl6/J was provided by Thomas Liehr (Institute of Human Genetics, Friedrich Schiller University, Germany).

### Preparation of leaf nuclei

Young leaf tissue of Arabidopsis and *Z. mays* was fixed in 4% and 2% (v/v) formaldehyde, respectively, using ice-cold Tris buffer [10 mM Tris–HCl, pH 7.5, 10 mM Na_2_-EDTA, 100 mM NaCl, 0.1% (v/v) Triton X-100] under vacuum for 5 min, followed by incubation for 25 min on ice without vacuum. The tissue was then rinsed twice in ice-cold Tris buffer for 5 min each. Subsequently, the tissue was chopped into 300–500 µl of ice-cold LB01 buffer [15 mM Tris–HCl, pH 7.5, 2 mM Na_2_-EDTA, 0.5 mM spermine, 80 mM KCl, 20 mM NaCl, 15 mM β-mercaptoethanol, and 0.1% (v/v) Triton X-100] using a fresh razor blade in a Petri dish as described by Doležel *et al.* (1989). The suspension was filtered through a 50 µm pore size mesh into a tube and, depending on the concentration of nuclei, either used undiluted or diluted up to 1:10 with LB01. Afterwards, 100 µl of suspension was spun onto glass slides using a Thermo Shandon Cytospin 3 centrifuge at 700 rpm for 5 min. The slides were either used immediately for labeling or stored in 1× phosphate-buffered saline (PBS) at 4 °C for a maximum of 24 h.

### Preparation of chromosomes

Root tip meristems were obtained from 3- to 5-day-old *Z. mays* and *V. faba* seedlings. The roots of both species were pretreated in 0.2 mM 8-hydroxyquinoline at room temperature (RT) for 3 h. Then, roots were fixed in 3:1 (ethanol: acetic acid) solution for 24 h at RT, washed twice in ice-cold ddH_2_O and 0.01 M citrate buffer (0.01 M sodium citrate and 0.01 M citric acid, pH 4.5–4.8), for 5 min each. Three to five root tips of *Z. mays* and *V. faba* were digested for 50 and 60 min, respectively, in 25–50 µl of enzyme mixture [0.7% (v/v) cellulase R10, 0.7% (v/v) cellulase, 1% (v/v) pectolyase, 1% (v/v) cytohelicase dissolved in 0.01 M citrate buffer] at 37 °C. The tubes with digested meristems were vortexed, and then after adding 600 µl of ddH_2_O at RT, they were centrifuged at 9391 *g* for 45 s. The supernatant was removed, and 600 µl 96% ethanol was added per tube and centrifuged at 11 363 *g* for 30 s. After the supernatant was discarded, the pellet was resuspended in 80–100 µl 96% ethanol. For the preparation of chromosome slides, 10 µl of cell suspension was dropped from a height of 10–15 cm onto a glass slide in a humid chamber placed on a hot plate at 55 °C. Then 25–50 µl of freshly prepared ethanol: acetic acid (3:1) was added to the glass slide at RT and air dried. Slides containing well-spread chromosomes were selected using a phase-contrast light microscope. Murine chromosomes were obtained from the skin of a laboratory house mouse strain C57Bl6/J and prepared as described (Potlapalli *et al.*, 2020).

### Plasmid construction

Vectors were prepared using Golden Gate cloning and the MoClo system, as detailed by Marillonnet and Grützner (2020) and Weber *et al.* (2011). In brief, individual components, including Sp-dCas9, 6× ALFA-tags, 3× enhanced green fluorescent protein (eGFP), mRuby, NbALFA, RPS5A promoter, Ubi4 promoter, Pea3A terminator, and rbcSE9 terminator, were separately amplified with primers (Supplementary Table S1) featuring overhangs containing *Bpi*I restriction sites (5ʹ-GAAGAC), 4-nt fusion sites, and *Bsa*I sites (5ʹ-GGTCTC) in opposite orientations. The Sp-dCas9, 3× eGFP, mRuby and U6-26 promoter with single-guide RNA (sgRNA) telomere sequences were amplified separately from previously described vectors Sa-dCas9-eGFP and Sp-dCas9-mRuby (Dreissig *et al.*, 2017). The 6× ALFA-tags were amplified from a synthesized DNA fragment obtained from Eurofins Genomics. Similarly, the NbALFA fragment was amplified from the Addgene vector pET51b(+)_eGFP_NbALFA 136626 (Farrants *et al.*, 2020). Next, these amplified components were individually cloned into level-0 vectors through Golden Gate cloning reactions using the *Bpi*I restriction enzyme, following the protocol described by Weber *et al.* (2011). The resulting cloning products were transformed into *Escherichia coli* and plated on LB plates supplemented with spectinomycin, isopropyl β-d-1-thiogalactopyranoside (IPTG), and X-gal. White colonies were selected, and the sequence was confirmed. Following verification, the transcript units, consisting of the promoter–Sp.dCas9–3×eGFP–6×ALFA-tag–terminator, and promoter–mRuby–NbALFA–terminator, were assembled into level-1 vectors using the Golden Gate cloning reaction using the *Bsa*I restriction enzyme. The cloned products were transformed into *E. coli* on LB plates containing ampicillin, IPTG, and X-gal, and white colonies were selected and confirmed by restriction digestion. Finally, Sp.dCas9–3×eGFP–ALFA-tag along with Arabidopsis telomere sgRNA under the U6 promoter, and mRuby–NbALFA expressing constructs were cloned separately into level-2 vector pICSL4723 using Golden Gate cloning with the *Bpi*I restriction enzyme. Following transformation into *E. coli* and selection on LB plates with kanamycin, white colonies were selected and confirmed through restriction digestion.

### Preparation of recombinant dCas9

The *Streptococcus pyogenes* dead version of the Cas9 gene, containing double nuclease mutations (D10A and H840A), was amplified from dCas9:3×PP7: GFP vector (Khosravi *et al.*, 2020b) and cloned into pET22b+ (Thermo Fisher Scientific) with a C-terminal hexahistidine affinity tag. For the construction of ALFA-fused dCas9 vectors, various copies of the ALFA-tag were amplified using PCR primers (Supplementary Table S1) from synthetic DNA fragments (Supplementary Table S2) that contained ALFA-tag sequences as a PCR template and then cloned at either the N- or the C-terminus of the dCas9 protein present in pET22b+. Plasmid encoding eGFP–NbALFA was obtained from Addgene (cat. no. 136626) (Farrants *et al.* 2020). These plasmids were then transformed into *E. coli* BL21 (DE3) and cultured in 2× TY medium at 37 °C until the OD_600_ reached ~0.5. Subsequently, the culture was induced with 0.5 mM IPTG and allowed to grow for 16 h at 18 °C. Following growth, cells were lysed in lysis buffer [50 mM NaH_2_PO_4_, 500 mM NaCl, 10% (v/v) glycerol, 10 mM imidazole, pH 8.0] containing 1 mg ml^−1^ lysozyme for 30 min on ice. The lysed culture was then snap-frozen in liquid nitrogen and immediately thawed at RT, followed by sonication on ice for four cycles of 30 s each at 50% intensity using a Vibra-Cell sonicater Model VC60 (Sonics & Materials Inc.). The resulting lysate was centrifuged at 4602 *g* for 20 min at 4 °C, and the supernatant was transferred to a Falcon tube containing 1 ml of PureCube 100 Ni-NTA Agarose (Cube Biotech, cat. no. 31103). The mixture was rotated at 4 °C for 90 min. The His-tagged dCas9 and eGFP–NbALFA proteins were subsequently purified by gravity flow chromatography using disposable polypropylene columns (Qiagen, cat. no. 34924). They were then eluted with elution buffer [50 mM NaH_2_PO_4_, 500 mM NaCl, 10% (v/v) glycerol, 250 mM imidazole, pH 8]. The purified proteins were stored at −20 °C for further use.

### sgRNA and RNP complex formation

To prepare guide RNA, lyophilized crRNA and tracrRNA-5ʹ-Atto550/biotin (Supplementary Table S2) were dissolved in nuclease-free duplex buffer (provided by Integrated DNA Technologies) to achieve a final concentration of 100 μM. Dissolved crRNA and tracrRNA were stored separately at −20 °C. sgRNA was formed by mixing 1 μl of 100 μM crRNA and 1 μl of 100 μM tracrRNA with 8 μl of nuclease-free duplex buffer in a PCR tube and denatured for 5 min at 95 °C in a PCR machine. The RNP complexes were assembled by mixing 1 μl (10 μM) of sgRNA, 1 μl of dCas9 (6.25 μM), 10 μl 10× Cas9 buffer [200 mM Hepes (pH 7.5), 1 M KCl, 50 mM MgCl_2_, 50% (v/v) glycerol, 10% (v/v) BSA, and 1% (v/v) Tween 20], 10 μl of (10 mM) DTT, and 80 μl of ddH_2_O, incubated at 26 °C for 10 min, and kept on ice until used.

### Standard CRISPR–FISH

One hundred microliters of 1× Cas9/1 mM DTT buffer was added to each slide for 2 min at RT, and then the buffer was removed by tilting the slides. Twenty to twenty-five microliters of RNP complex was added per slide, covered with Parafilm, and incubated at 37 °C for 1 h in a humid chamber. After incubation, slides were washed in 1× PBS for 5 min on ice and post-fixed with 4% (v/v) formaldehyde in 1× PBS for 5 min at RT. Then, slides were washed with 1× PBS at RT and dehydrated in an ethanol series (70%, 90%, and 96%) for 2 min each. Finally, slides were air-dried in the dark and counterstained with 8 µl of (0.5 µg ml^−1^) 4ʹ,6-diamidino-2-phenylindole (DAPI) in antifade mounting medium (Vector Laboratories, Burlingame, CA, USA) and stored at 4 °C for microscopic evaluation.

### Indirect CRISPR–FISH method with streptavidin fluorescein isothiocyanate

The RNP complex was prepared as described above using the dCas9 protein, target-specific crRNA and 3ʹ-biotin-labeled tracrRNA. Slides were washed with 0.2% Triton X-100 in Tris–HCL (pH 9.0) at 37 °C for 30 min and later in 1× PBS twice for 5 min each. The RNP complex was applied, and slides were post-fixed in a similar manner to standard CRISPR–FISH as described above. Then, slides were incubated with 100 μl of blocking solution [4% (v/v) BSA, 0.1% (v/v) Tween 100, 1× PBS] under Parafilm for 30 min at RT, washed twice for 5 min each in 1× PBS, incubated with 50 μl of streptavidin-conjugated fluorescein isothiocyanate (FITC) (Sigma-Aldrich; 1:100 diluted) in 1% (v/v) BSA in 1× PBS for 1 h at 37 °C under Parafilm in a humid chamber, followed by washing thrice for 5 min each in 1× PBS in the dark and then dehydrated in an ethanol series (70%, 90%, and 96%) for 2 min each. Finally, slides were air-dried in the dark and counterstained with 8 µl of DAPI in antifade mounting medium and stored at 4 °C for microscopic evaluation.

### CRISPR–FISH with ALFA-tagged dCas9

RNP complexes were prepared using the dCas9 protein fused with 1–24 copies of the ALFA-tag, target-specific crRNA and Atto550 tracrRNA as described above. Slides were washed with 0.2% (v/v) Triton X-100 in Tris–HCl (pH 9.0) at 37 °C for 30 min, followed by two 5 min washes in 1× PBS. The RNP complex was applied similarly to indirect CRISPR–FISH. After post-fixation, slides were blocked with 100 μl of 4% (v/v) BSA in 1× PBS at RT in a humid chamber and then incubated with 50 μl of FluoTag-X2 anti-ALFA conjugated with Atto488 (FluoTag-X2 anti-ALFA, cat. no. N1502, NanoTag Biotechnologies GmbH, diluted 1:500) or 50 μl of eGFP–NbALFA (1 μM) in 1% (v/v) BSA in 1× PBS for 1 h at 37 °C under Parafilm in a humid chamber, followed by three washes in 1× PBS for 5 min each in the dark, and further dehydration in an ethanol series (70%, 90%, and 96%) for 2 min each. Finally, slides were air-dried in the dark and counterstained with 8 µl of DAPI in antifade mounting medium and stored at 4 °C for microscopic evaluation.

In case of using a recombinant sdAb anti-ALFA rabbit Fc-fusion (Minibody, N1583, Nanotag Biotechnologies GmbH), after blocking and washing with the 1× PBS in dark, 50 μl minibody (diluted 1:500) in 1% (v/v) BSA in 1× PBS was added and incubated for 1 h at 37 °C under Parafilm in a humid chamber, followed by three washes in 1× PBS for 5 min each in the dark. Slides were then probed with 50 μl of secondary goat anti-rabbit Alexa 488 (1:100 diluted) for 1 h at 37 °C under Parafilm in a humid chamber, followed by three washes in 1× PBS for 5 min each in the dark. They were later dehydrated, air-dried and counterstained as described above.

### Tyramide-based signal amplification of CRISPR–FISH signals

For tyramide-based amplification, first CRISPR–FISH was performed as described above using 3ʹ-biotinylated tracrRNA till post-fixation and washing for 5 min in 1× PBS at RT. Tyramide signal amplification was performed using the Biotin XX Tyramide SuperBoost Kit, Streptavidin (cat. no. B40931, Thermo Fisher Scientific), according to the manufacturer’s instructions. Afterwards, slides were blocked with 100 µl of blocking buffer for 30 min at RT and incubated with 100 µl of HRP-conjugated streptavidin for 1 h at RT in a humid chamber. After three washes in 1× PBS for 5 min each at RT, slides were incubated with 100 µl of tyramide working solution in a humid chamber for 10 min at RT. Then, the reaction was terminated by adding 100 µl of reaction stop reagent for 1 min at RT. After three washes in 1× PBS for 5 min each at RT, slides were incubated each with 50 µl of streptavidin-conjugated FITC (cat. no. S3762, Sigma-Aldrich) diluted 1:50 in (v/v) 1% BSA/1× PBS per slide at 37 °C for 1 h in a humid chamber. Subsequently, the slides were washed thrice in 1× PBS for 5 min each in dark, dehydrated, and counterstained as described for standard CRISPR–FISH.

### Transient transformation of *N. benthamiana* with a live imaging construct

The level-2 expression constructs Sp.dCas9–3×eGFP–ALFA-tag (Supplementary Fig. S1A), and mRuby–NbALFA (Supplementary Fig. S1B) were individually introduced into the *Agrobacterium tumefaciens* strain GV3101 through electroporation. The agrobacteria carrying the expression vectors were cultured overnight at 28 °C in YEB medium supplemented with 100 mg ml^−1^ kanamycin and 50 mg ml^−1^ rifampicin. Transient transformation of *N. benthamiana* was conducted following the method described by Phan and Conrad (2016). For co-transformation experiments, bacterial cultures with an equivalent OD_600_ (0.6) were mixed in a 1:1 ratio prior to the transformation process. Plant samples were analysed by microscopy after 48 h of infiltration.

### Immunostaining and fluorescence *in situ* hybridization

After 48 h of infiltration, a 1 cm² piece of leaf tissue expressing dCas9–3×eGFP–ALFA-tag along with telomere sgRNA was fixed in 4% (v/v) formaldehyde, as described above. Nuclei were extracted using LB01 buffer, filtered, and spun onto a glass slide with a CytoSpin3 (Shandon) at 400 rpm for 5 min. To confirm the specificity of live signals, we merged CRISPR imaging with FISH. The CRISPR signal intensity was enhanced through indirect immunostaining of eGFP, utilizing a monoclonal anti-GFP antibody (GFP Antibody Dylight™ 488 Conjugated, cat. no. 200-341-215, Rockland) in a 1:2500 dilution according to Ishii *et al.* (2015). Then, slides were washed twice in 1× PBS for 5 min each and fixed in 3:1 (ethanol: acetic acid) solution for 24 h at RT in the dark. Subsequently, slides were dehydrated in an ethanol series (70%, 90%, and 96%) for 2 min each and air-dried. Afterwards, an overnight pre-hybridization was performed at 37 °C by adding 15 µl of FISH hybridization solution [50% (v/v) formamide, 10% (v/v) dextran sulfate in 2× saline sodium citrate (SSC; 0.30 M sodium citrate, 0.030 M NaCl, pH 7.0)]. Slides were then washed twice in 2× SSC for 5 min each and subsequently dehydrated in an ethanol series for 2 min each and air-dried. After, DNA denaturation was carried out in 0.2 M NaOH in 70% ethanol at RT for 7 min followed by sequential dehydration in an ethanol series for 2 min each, and then air-dry in the dark. Subsequently, 14 µl of FISH hybridization solution along with 1 µl of 5ʹ-Cy5-labeled telomere oligonucleotide probe (Supplementary Table S2) was added per slide and hybridized at 37 °C overnight in a humid chamber. Slides were washed twice in 2× SSC for 5 min each in dark and then dehydrated in an ethanol series for 2 min each. Finally, slides were air-dried in dark, counterstained with 8 µl of DAPI in antifade mounting medium, and stored at 4 °C for microscopy.

### Detection of live telomere signals with a minibody

For minibody-based detection of CRISPR–ALFA-tag live telomere signals, nuclei slides were prepared from *N. benthamiana* leaves expressing dCas9–3×eGFP–ALFA-tag along with telomere sgRNA as described above. Slides were then washed twice in 1× PBS for 5 min each and blocked and detected with sdAb anti-ALFA rabbit Fc-fusion, and subsequently detected with 50 μl of secondary goat anti-rabbit Alexa Fluor 555 antibody as described above.

### Fluorescence microscopy

Microscopy images were captured using an epifluorescence microscope (Olympus BX61) equipped with a cooled charge-coupled device (CCD) camera (Orca ER; Hamamatsu). For initial observation of successful transformation, *in vivo* fluorescence signals were analysed by cutting a piece of the infiltrated leaf and utilizing a ×60/NA 1.2 water objective. All fluorescence images were initially captured in grayscale and later pseudo-colored using ImageJ software for analysis and visualization. Live imaging of probes was examined using a Zeiss LSM780 confocal laser scanning microscope (Carl Zeiss, Jena, Germany) with a C-apochromat ×40/NA 1.2 water objective. Nuclear GFP and mRuby signals were analysed using appropriate filters. Employing zoom 4.0, image size was 512 × 256 pixels, pixel dwell time 1.56 µs, and pinhole set at 32 µm. For time series, Z-stacks were recorded every 30 s.

### CRISPR–FISH signal quantification

For the quantification of CRISPR–FISH signals, a standardized protein concentration of 1 mg ml^−1^ for all dCas9–ALFA variants fused with various copies of the ALFA-tag was applied. Subsequently, all fluorescence images were captured with a CCD camera using an exposure time of either 100 ms or 50 ms, and ImageJ software was employed to measure the signal intensities. In brief, individual nuclei were defined using the threshold function of ImageJ. Measurements of the corresponding signals, including area, mean, minimum and maximum grey values, and integrated density, were then recorded for both green (anti-rabbit antibody Alexa488—minibody) and red (tracrRNA labeled with Atto550) channels within the identified nuclear regions. Additionally, equivalent measurements were taken inside the nuclear areas, excluding centromere signals in both red and green channels, to calculate the nuclear background. Subsequently, the nuclear background was subtracted from the actual target signals to determine the green and red signal intensities. To compensate for potential slide-to-slide and experiment-to-experiment variations as well as different exposure times, the red tracrRNA signal was considered as reference to calculate the relative green fluorescence intensities as green/red signal ratios. Box plots were prepared using an online tool BoxPlotR, (http://shiny.chemgrid.org/boxplotr/).

### Statistical analysis

To evaluate the impact of the ALFA-tag copy numbers on the relative signal intensities we performed Kruskal–Wallis one way analysis of variance (ANOVA) on ranks and subsequently a pairwise multiple comparison (Dunn’s method). The significance was tested at *P*<0.01 level. The difference in signal intensity between 6× and 6×GS was tested for significance employing the Mann–Whitney rank sum test. To evaluate the effect of the ALFA-tag copies distribution (single-sided versus double-sided) in relation to the copy number, a two-way ANOVA and a subsequent pairwise multiple comparison (Holm–Šidák method) was performed. The significance was tested at *P*<0.01 level. For all analyses the program SigmaPlot 12 (Systat Software, Inc., 2012) was used.

## Results

### Establishing an ALFA-tag-assisted CRISPR–FISH system for labeling DNA repeats on fixed samples with an ALFA-specific nanobody

**Fig. 1.**
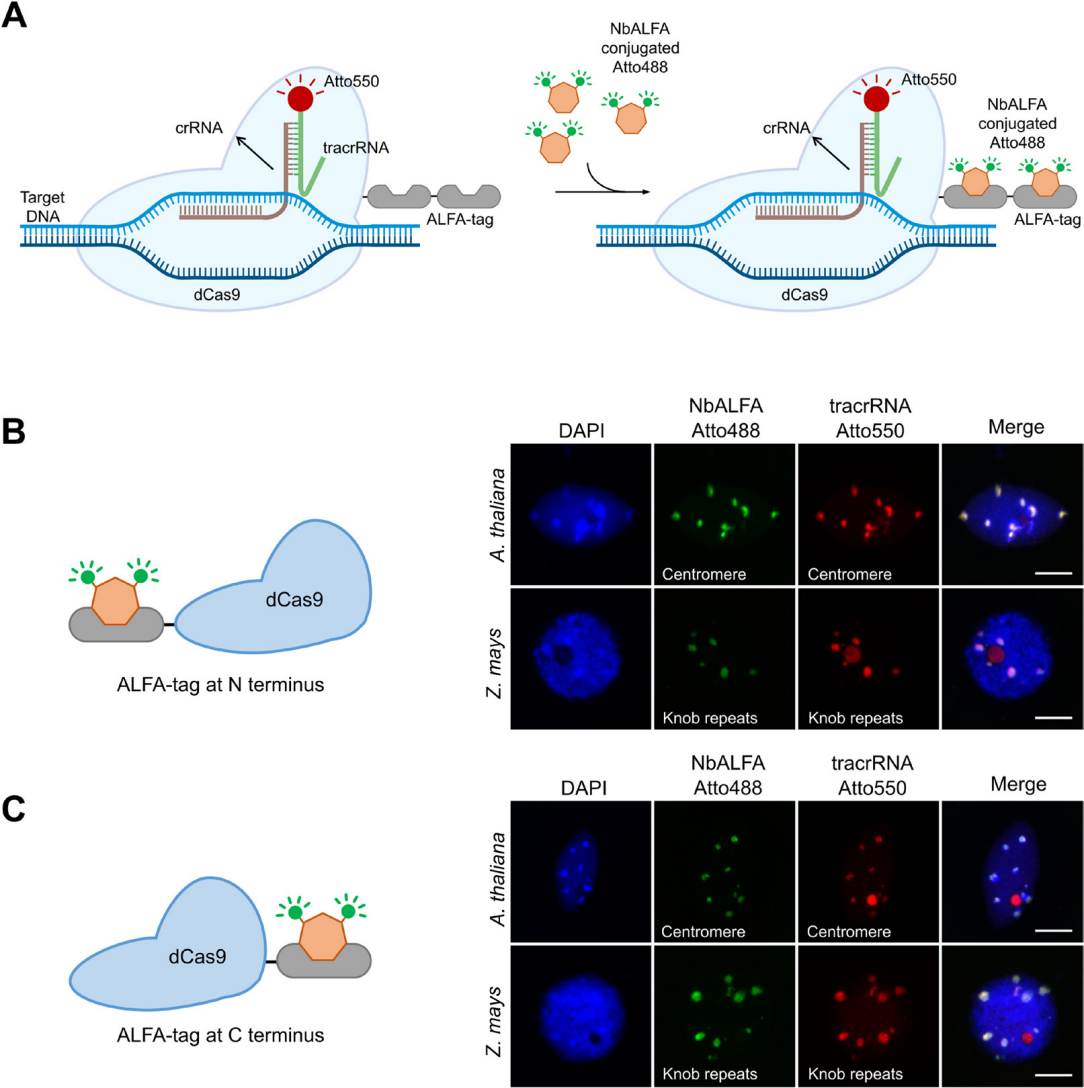
Visualization of DNA repeats using dCas9–ALFA-tag and NbALFA combination. (A) Schematic representation of CRISPR–FISH in the combination of ALFA-tag and NbALFA. A dCas9 protein fused with ALFA-tags, in conjunction Atto550-tagged bipartite gRNA, is used to label DNA repeats. Covalent detection of ALFA-tagged dCas9 with NbALFA conjugated to Atto488. (B, C) Sketch illustrating the dCas9 protein fused with the ALFA-tag at either the N-terminus (B) or the C-terminus (C). On the right side, visualization of Arabidopsis centromere and *Z. mays* knob repeats in formaldehyde fixed nuclei using modified dCas9 proteins with a single ALFA-tag at either the N-terminus (B) or the C-terminus (C). Green signals represent NbALFA Atto488, and red signals correspond to Atto550-tagged tracrRNA. Nucleus is counterstained with DAPI (in blue). Scale bars: 10 µm. crRNA, CRISPR RNA; DAPI, 4ʹ,6-diamidino-2-phenylindole; FISH, fluorescence *in situ* hybridization; gRNA, guide RNA; tacrRNA, trans-activating CRISPR RNA.

To establish a CRISPR–FISH ALFA-tag system for amplifying the target signal intensity, we constructed a dCas9 protein fused with variable numbers of ALFA-tags. The ALFA-tag can be detected efficiently and covalently by an ALFA-specific nanobody (NbALFA) conjugated with various organic fluorescent dyes (Götzke *et al.*, 2019). For method development, we utilized ALFA-tagged dCas9 in conjunction with Atto550-tagged bipartite gRNA and NbALFA conjugated with Atto488 (Fig. 1A). First, to assess whether the ALFA-tag would impact the DNA binding ability of dCas9, we employed the dCas9 protein fused to a single copy of the ALFA-tag at either the N- (Fig. 1B) or the C-terminus (Fig. 1C). Centromere and knob repeat-specific gRNAs were used for CRISPR–FISH on nuclei of Arabidopsis and *Z. mays*, respectively (Supplementary Table S2). Co-localization of red tracrRNA and green NbALFA signals of either N- or C-terminus ALFA-tagged dCas9 illustrated the compatibility of the ALFA-tag with the dCas9 protein function when placed at either side of the protein (Fig. 1B, C). Additionally, dCas9 protein with ALFA-tag fused on the N-terminus was successfully used to label the knob, *Fok*I repeats, and major satellite repeats of ethanol: acetic acid (3:1)-fixed *Z. mays*, *V. faba*, and mouse chromosomes, respectively (Supplementary Fig. S2).

To assess the potential enhancement of Arabidopsis centromere repeat signal intensities by increasing the number of ALFA-tag copies, we engineered dCas9–ALFA-tag proteins, which were fused with either three copies of the ALFA-tag at the N-terminus or three ALFA copies each on both N- and C-termini of the dCas9 protein (Supplementary Fig. S3A). However, increasing the number of ALFA-tags did not result in an obvious signal increase of NbALFA conjugated Atto488. Additionally, to evaluate whether the recombinantly purified NbALFA–eGFP could produce stronger signal intensities than NbALFA–Atto488, we labeled the Arabidopsis centromere repeats with dCas9 fused with 6× copies (3 + 3×) of the ALFA-tags in combination with NbALFA–eGFP. In this case no obvious enhancement in signal intensities was observed (Supplementary Fig. S3B).

### Enhanced centromere signal intensity with the dCas9–ALFA system in combination with a minibody

**Fig. 2.**
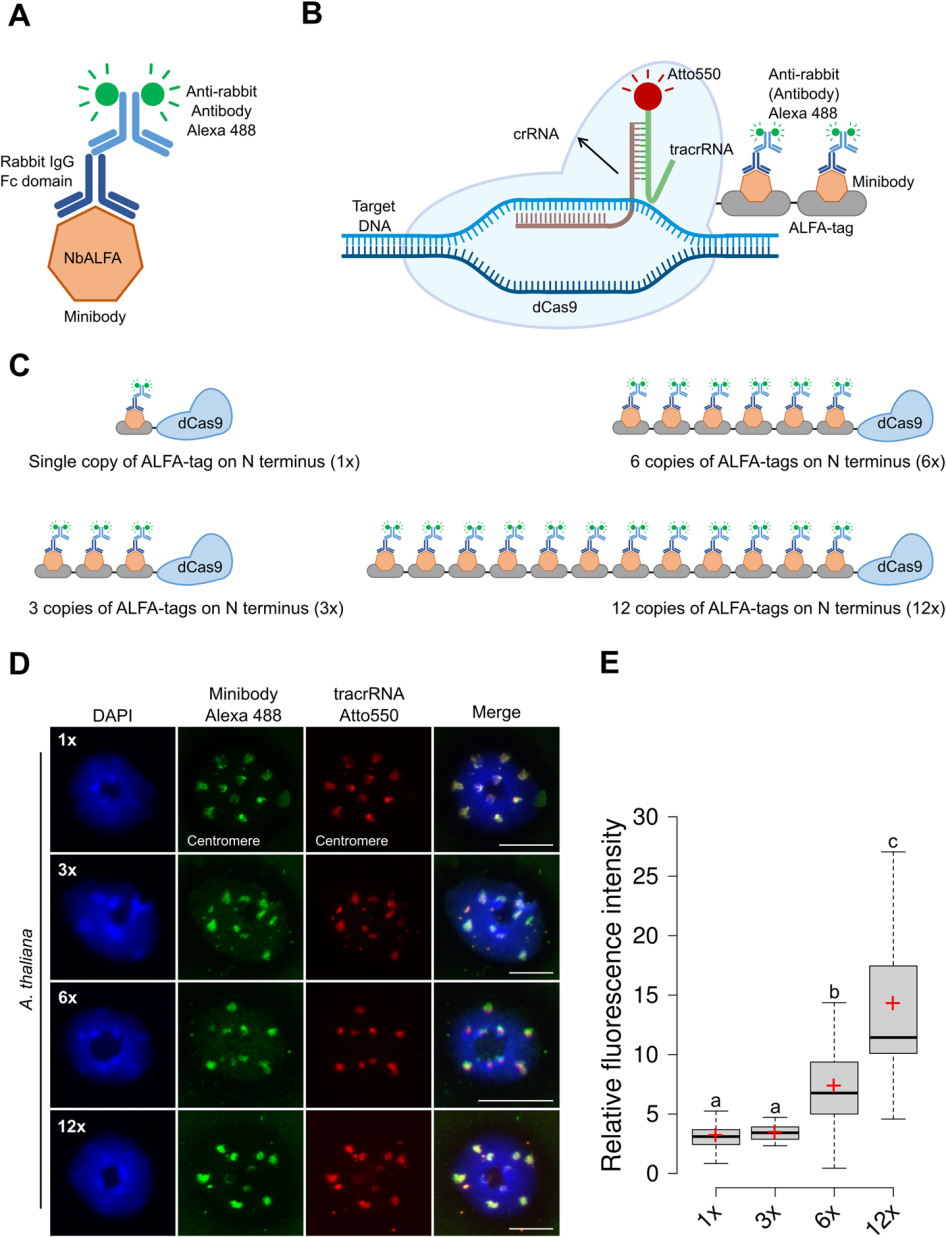
Enhanced centromere labeling intensities using dCas9–ALFA-tag and minibody combination. (A) Sketch of the minibody having anti-ALFA single-domain genetically fused to rabbit Fc domain. (B) Schematic representation of CRISPR–FISH in the combination of ALFA-tag and minibody. A dCas9 protein fused with ALFA-tags, in conjunction Atto550-tagged bipartite gRNA, is used to label DNA repeats and later probed with a minibody and subsequently detected with anti-rabbit Alexa 488. (C) Sketch illustrating the dCas9 protein fused with 1× (upper left), 3× (lower left), 6× (upper right) or 12× (lower right) ALFA copies at the N-terminus of dCas9 protein. (D) Visualization of centromere repeats using dCas9–ALFA-tag proteins detected with minibody and anti-rabbit Alexa 488. Green signals represent anti-rabbit Alexa 488, and red signals correspond to Atto550-tagged tracrRNA. Nuclei are counterstained with DAPI (in blue). Scale bars: 10 µm. (D) Boxplots showing the relative fluorescence intensity from centromere foci generated by different dCas9 ALFA copy variants in conjunction with the minibody. The numbers 1×, 3×, 6×, and 12× represent varying ALFA-tag copy numbers fused to the dCas9 protein at the N-terminus. Each analysis comprises at least 50 nuclei. The boundaries of the box indicate the 25th and 75th percentile, the error bars the 10th and 90th percentile, the black line the median and the plus symbol the mean. Different letters above the boxes indicate significant differences between groups (*P*<0.01). crRNA, CRISPR RNA; DAPI, 4ʹ,6-diamidino-2-phenylindole; FISH, fluorescence *in situ* hybridization; gRNA, guide RNA; tacrRNA, trans-activating CRISPR RNA.

To address the lack of signal intensity increase resulting from the combination of NbALFA with increasing ALFA copies, we introduced a minibody into our experimental approach. This minibody is a single-domain antibody (sdAb) consisting of an anti-ALFA single-domain genetically fused to the IgG Fc domain of a commonly used host species. The minibody provides a high affinity for the ALFA-tag and exhibits attributes similar to a conventional IgG-type antibody. In our approach, we employed a minibody genetically fused to a rabbit Fc domain (Fig. 2A). In practice, following the binding of dCas9–ALFA to the target DNA, the minibody detects the ALFA-tagged RNP complex and is subsequently visualized by an Alexa 488-conjugated anti-rabbit antibody (Fig. 2B). To assess its functionality, we visualized the centromere repeat on fixed Arabidopsis nuclei using a recombinant dCas9–ALFA-tag protein fused with one ALFA-tag (Fig. 2C). We obtained a clear centromere labeling (Alexa 488) co-localizing with tracrRNA (Atto550), confirming the specificity of the minibody (Fig. 2D).

To assess the potential to enhance the signal intensity, we next applied dCas9–ALFA fusion proteins with 3, 6, or 12 ALFA-tag copies (Fig. 2C). Remarkably, notable variations in minibody signal intensities were observed among different ALFA copy numbers (Fig. 2D), prompting a quantitative analysis. To confirm that these differences indeed resulted from the increase of ALFA copies, red signal intensities from the Atto550-conjugated tracrRNA were used as references to calculate the relative green (Alexa 488) fluorescence intensities (see ‘Materials and methods’). While we found for the dCas9–ALFA constructs with one and three ALFA-tag copies at the N-terminus only a low and not significant increase (means: 3.22 and 3.47, respectively), six copies resulted in a significant increase (mean: 7.37) (Fig. 2E) corresponding to a roughly 2-fold change; 12× ALFA copies doubled the signal intensity further (mean: 14.31). In summary, these results confirm the consistent ability of the minibody to enhance CRISPR–FISH signal intensity with increased ALFA copies.

### Potential enhancement of CRISPR–FISH signal intensity by incorporating linkers between the ALFA-tag copies or separating them to both ends of the dCas9 protein

**Fig. 3.**
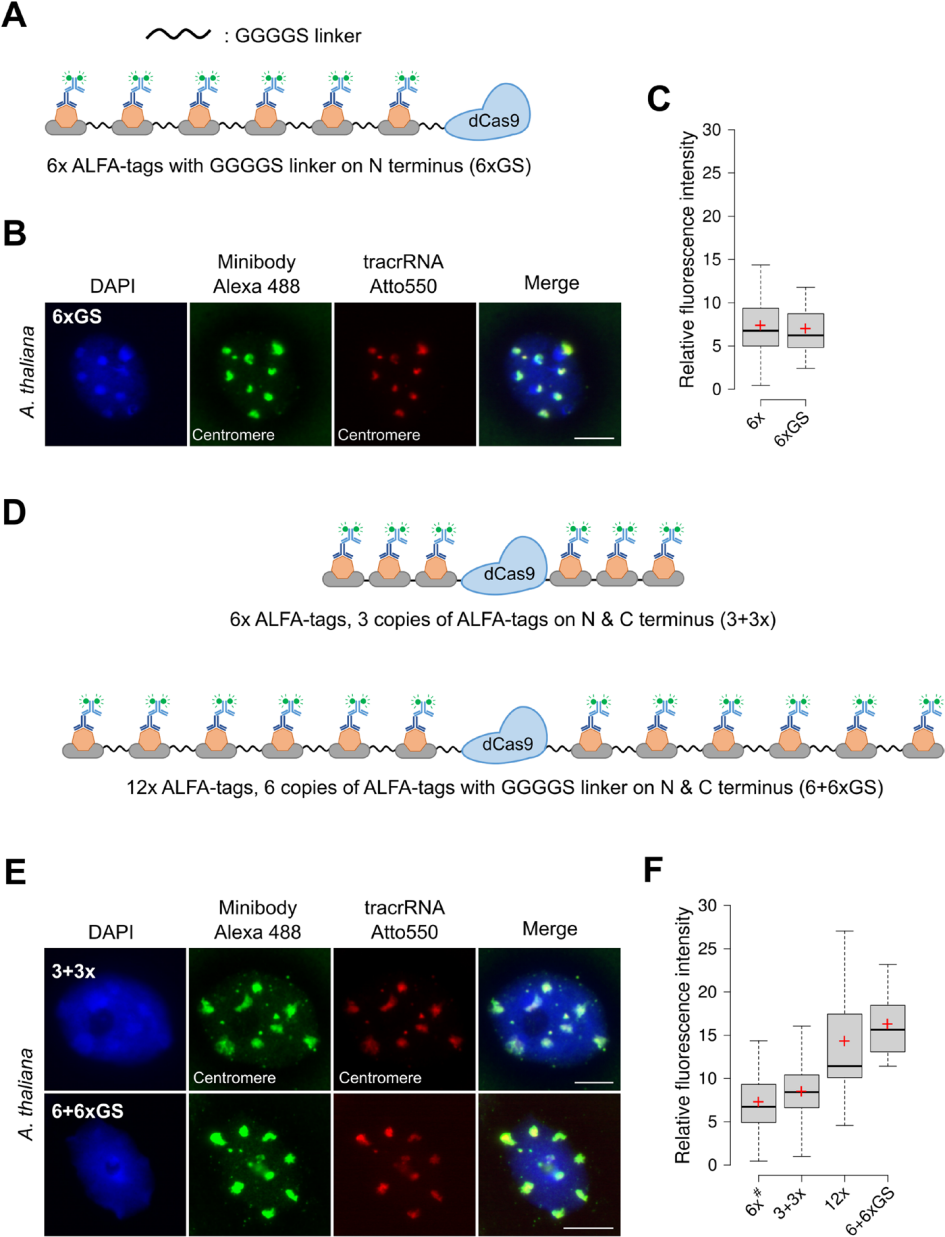
Potential increase of signal intensities by using linkers between ALFA-tag copies and separating the ALFA-tag copies to both sides of dCas9. (A) Sketch illustrating the dCas9 protein fused with 6× ALFA copies on N-terminus separated by glycine linkers (GGGGS). (B) Visualization of centromere repeats using the 6× ALFA copies, separated by glycine linkers (GGGGS) and detected with minibody and anti-rabbit Alexa 488. (C) Boxplots showing the relative fluorescence intensity from centromere foci generated by 6× ALFA-tags separated by glycine serine linkers (6×GS) in comparison with the 6× construct without linkers (6×) in conjunction with the minibody. Analysis of 6× comprises 50 nuclei, and for 6×GS 25 nuclei were analysed. The difference between both groups was statistically not significant (*P*=0.861). (D) Sketch illustrating the dCas9 proteins fused with either 3× ALFA copies (upper) or 6× ALFA copies (lower) on each N- and C-terminal end of dCas9 protein. In the latter, the ALFA copies are separated by GS linkers (GGGGS). (E) Visualization of centromere repeats using the 3 + 3× ALFA copies and 6 + 6×GS ALFA copies separated by GS linkers and detected with minibody and anti-rabbit Alexa 488. In (B, E), green signals represent anti-rabbit Alexa 488, and red signals correspond to Atto550-tagged tracrRNA. The nucleus is counterstained with DAPI (in blue). Scale bars: 10 µm. (F) Boxplots showing the relative fluorescence intensity from centromere foci generated by 3 + 3× and 6 + 6×GS ALFA-tags in comparison with constructs with identical copy numbers fused to only the N-terminus (6×^#^ and 12×). 6×^#^ comprises data from 6× and 6×GS, since we detected no significant difference between these two variants. Per construct at least 25 nuclei were evaluated. Two-way analysis of variance (ANOVA) followed by pairwise multiple comparison (Holm–Šidák method) indicated a significant effect of both copy number (*P*<0.001) and distribution of the copies (single-sided versus double-sided; *P*<0.01) on the signal intensity. The boundaries of the boxes in (C, F) indicate the 25th and 75th percentile, the error bars the 10th and 90th percentile, the black line the median and the plus symbol the mean. Sketches illustrating the variants compared here are shown in Fig. 2C. DAPI, 4ʹ,6-diamidino-2-phenylindole; tacrRNA, trans-activating CRISPR RNA.

Using linker sequences between independent fusion proteins enhances their stability, activity, and independent functionality (Chen *et al.*, 2013; Guo *et al.*, 2021). Glycine-rich linkers, which are naturally occurring and non-interfering with protein function, have proven to be particularly useful in this regard (Reddy Chichili *et al.*, 2013). To assess the impact of incorporating linkers between individual ALFA-tags on the minibody signal intensity, we generated dCas9 proteins fused with glycine linker (GGGGS), allowing for a distinct separation of individual ALFA-tag copies (Fig. 3A). These modified proteins were successfully utilized to label the Arabidopsis centromere repeats, facilitating subsequent quantitative analysis (Fig. 3B). dCas9 proteins fused with 6×GS (six copies) ALFA-tag copies separated by linkers displayed a relative signal intensity (mean: 7.00) very similar to the one obtained with construct without linkers (6×; mean: 7.37) (Fig. 3C). A comparison of both data sets revealed no statistically significant difference indicating no impact of the GS linkers on the signal intensity. Therefore, we pooled the data obtained for 6× and 6×GS for later comparisons (6×^#^).

Next, we included constructs with 6×, 12×, and 24× ALFA-tag copies in each of which half of them were fused to the N- and half to the C-terminus (Fig. 3D; Supplementary Fig. S4A). In all cases the centromere clusters of Arabidopsis were successfully labeled (Fig. 3E), but labeling with the 24× ALFA copies was weaker and showed an increased level of background signals (Supplementary Fig. S4B) and was therefore excluded from further analysis. Quantitative analysis revealed for the two remaining cases, 3 + 3× and 6 + 6×GS, mean values (8.49 and 16.29) higher than those of the corresponding constructs with the same copy numbers fused to the N-terminus only (6×^#^: 7.28 and 12×: 14.31) (Fig. 3F). Performing a two-way ANOVA and a subsequent pairwise multiple comparison (Holm–Šidák method) indicated significant differences for the factor ‘copy number’ independent of the distribution of the copies (single-sided: 6× and 6×GS versus 12× as well as double-sided: 3 + 3× versus 6 + 6×GS). Similarly, we obtained significant differences for the factor ‘copy distribution’ (single-sided, double sided) for six copies (6× and 6×GS versus 3 + 3×) as well as for 12 copies (12× versus 6 + 6×GS). When the ALFA-tags are located on both sides, the dCas9 protein itself might work as a big spacer allowing a better detection of the ALFA tag copies.

### Live imaging of telomere repeats in *N. benthamiana* with dCas9–ALFA tag

**Fig. 4.**
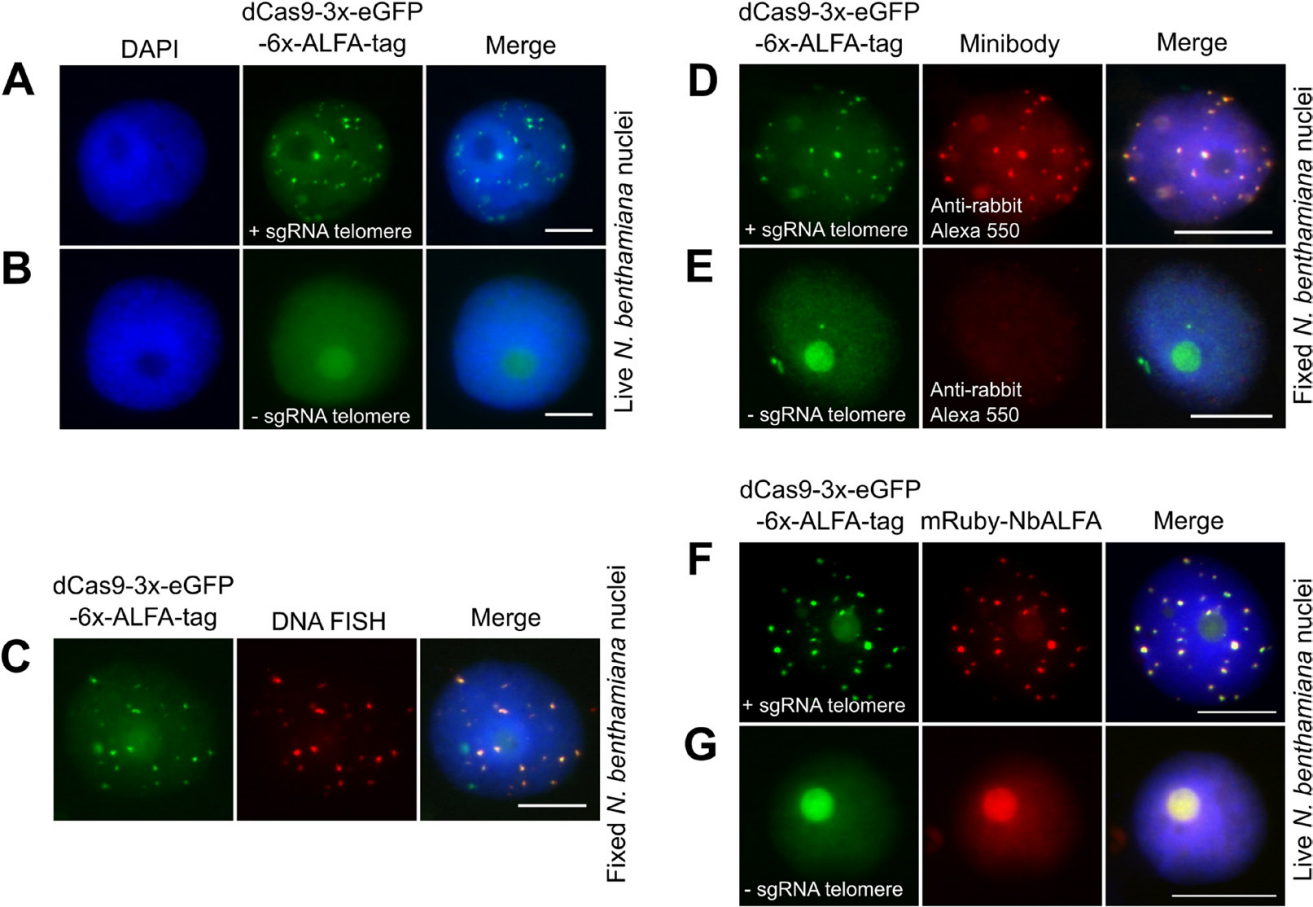
Live imaging of telomere repeats with the dCas9–ALFA-tag system. (A) Sp.dCas9–3×eGFP–ALFA-tag and sgRNA telomere were used for live imaging of telomeres in *N. benthamiana* leaf cells during interphase. (B) As a negative control, the telomere sgRNA was omitted. (C) The specificity of live telomere labeling using the dCas9–eGFP–ALFA-tag was confirmed by co-localization with DNA FISH. (D) Detection of telomere labeling of Sp.dCas9–3×eGFP–ALFA-tag with minibody and staining with anti-rabbit Alexa 488 resulted in telomere-like signals. (E) As a negative control, a minibody was applied to the leaf nuclei without expressing telomere sgRNA. (F) Dual labeling of telomeres in *N. benthamiana* using Sp.dCas9–3×eGFP–ALFA-tag and mRuby–NbALFA yielded green and red signals, with evident co-localization. This confirms the specificity of NbALFA towards ALFA-tagged dCas9. (G) A similar experiment was conducted without expressing telomere sgRNA as a negative control. Nuclei were counterstained with DAPI (in blue). Scale bars: 10 µm. DAPI, 4ʹ,6-diamidino-2-phenylindole; eGFP, enhanced green fluorescent protein; FISH, fluorescence *in situ* hybridization; sgRNA, single-guide RNA.

Fusing fluorescent proteins directly to dCas9 successfully labeled telomeres in live leaf cells of *N. benthamiana* (Dreissig *et al.*, 2017; Khosravi *et al.*, 2020b). To evaluate the *in vivo* labeling potential of the dCas9–ALFA-tag for DNA repeats, we fused ALFA-tags, to the *Staphylococcus pyogenes* dCas9 protein. To maintain construct compactness and alleviate the load on dCas9, we incorporated six ALFA-tag copies separated by glycine linkers (GGGGS) at the C-terminal end of the dCas9 protein fused with 3× copies of eGFP and utilized a previously reported telomere-targeting sgRNA under the Arabidopsis U6-26 promoter. The Sp.dCas9-3×eGFP–ALFA-tag, along with telomere sgRNA was incorporated into the plant expression vector (Supplementary Fig. S1A) and then transiently infiltrated into *N. benthamiana* leaves. After 48 h, telomere-like fluorescent puncta were observed, along with non-specific background labeling of the nucleus (Fig. 4A; Supplementary Video S1). As expected, no telomere-specific labeling was observed when infiltrated with a similar construct lacking the telomere sgRNA, resulting in only non-specific labeling of the nucleolus (Fig. 4B). The specificity of the *in vivo* dCas9–ALFA-tag telomere labeling was confirmed by clear co-localization of red telomere FISH signals with green signals caused by dCas9–3×eGFP–ALFA tag with telomere-specific sgRNA (Fig. 4C). As addional control, dCas9–ALFA tag telomere labeling was detected using the minibody, subsequently labeled with anti-rabbit Atto488 (Fig. 4D). Notably, we did not detect telomere signals with minibody in nuclei expressing the dCas9–3×eGFP–ALFA tag without telomere gRNA (Fig. 4E).

To assess *in vivo* recruitment of NbALFA by dCas9–ALFA tags for telomere labeling, we employed recombinant NbALFA fused with mRuby, regulated by the RPS5A promoter. Both vectors were separately introduced into *N. benthamiana* leaves: (i) Sp.dCas9–3×eGFP–ALFA-tag with telomere gRNA (Supplementary Fig. S1A) and (ii) mRuby–NbALFA (Supplementary Fig. S1B). After 48 h, both dCas9–3×eGFP–ALFA tag and mRuby–NbALFA led to dual labeling of telomeres in green and red (Fig. 4F; Supplementary Video S2). Omitting gRNA resulted in only green and red labeling of the nucleus without a telomere-specific labeling (Fig. 4G). Collectively, these findings showcase the targeted *in vivo* recruitment of mRuby–NbALFA by dCas9–3×eGFP–ALFA tags, accomplishing dual labeling of telomeres and confirming specificity through signal co-localization.

### Tyramide signal amplification in combination with CRISPR–FISH

**Fig. 5.**
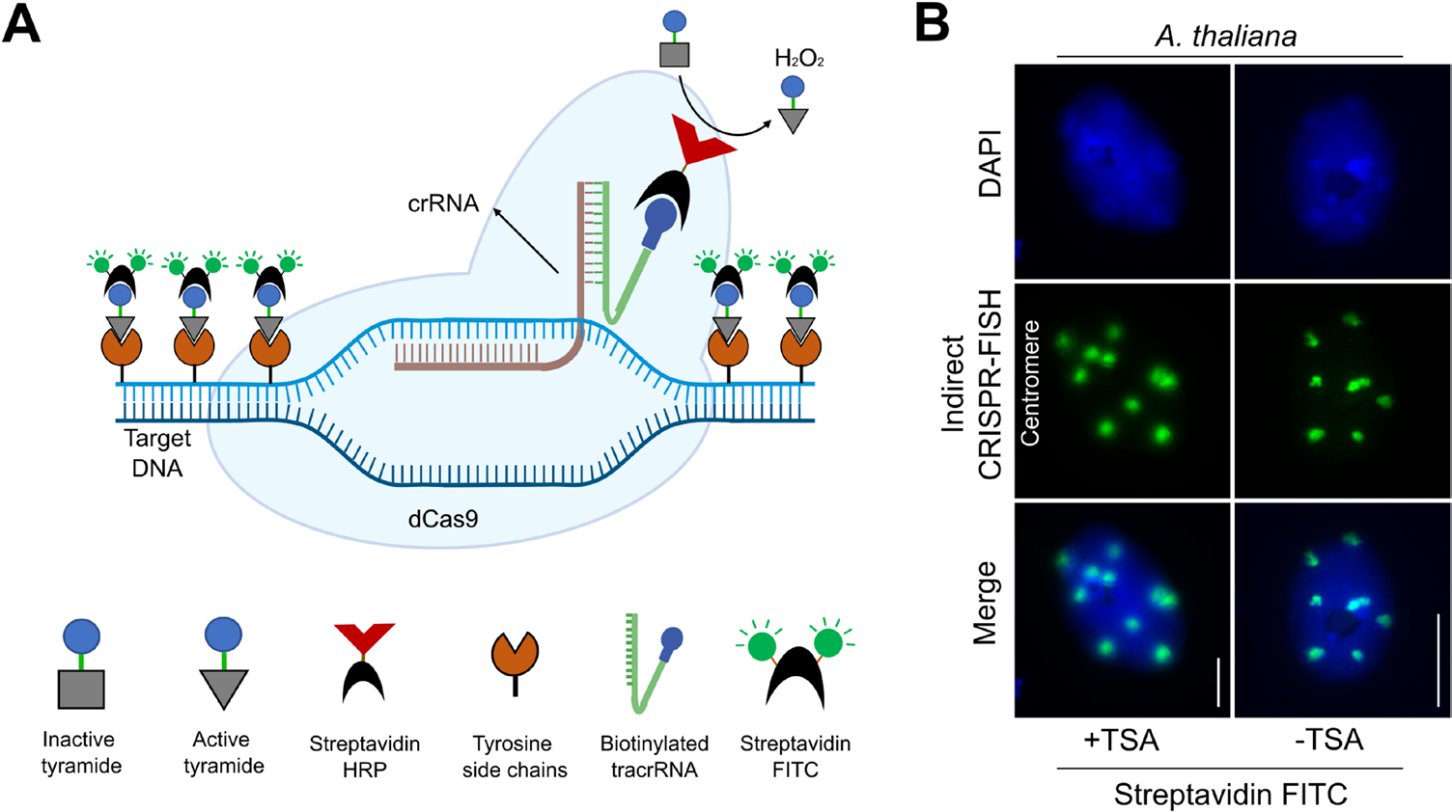
Tyramide signal amplification system in combination with indirect CRISPR–FISH. (A) Schematic illustration of the method. A dCas9 protein in combination with biotin-tagged bipartite gRNA is used to label DNA repeats. The horseradish peroxidase, conjugated to streptavidin, in the presence of H_2_O_2_ catalyses the conversion of labeled biotin tyramide into a reactive radical. The biotin tyramide radical then covalently binds to nearby tyrosine residues, and later application of streptavidin–FITC provides high-density labeling. (B) Labeling of Arabidopsis centromeres using indirect CRISPR–FISH after TSA application (left). As a negative control (right), centromere visualized with streptavidin–FITC without TSA application. Nuclei were counterstained with DAPI (in blue). Scale bars: 10 µm. crRNA, CRISPR RNA; DAPI, 4ʹ,6-diamidino-2-phenylindole; FISH, fluorescence *in situ* hybridization; FITC, fluorescein isothiocyanate; gRNA, guide RNA; HRP, horseradish peroxidase; TSA, tyramide-assisted amplification.

TSA is a sensitive *in situ* method utilizing HRP-catalysed tyramide deposition near the target nucleic acid sequence or protein (Raap *et al.*, 1995). Typically, it involves the use of HRP-conjugated antibodies or streptavidin. To combine TSA with CRISPR–FISH, we utilized a biotinylated tracrRNA for the indirect labeling of DNA repeats with a streptavidin conjugate. This biotin-conjugated tracrRNA enabled the combination of TSA with indirect CRISPR–FISH. In this approach, the RNP complex labels the target DNA first, followed by the application of streptavidin HRP to interact with the biotinylated tracrRNA. Subsequent incubation with the biotin tyramide working solution results in the deposition of biotin-tagged tyramide in the proximity of target DNA sequences. Finally, the application of streptavidin–FITC allows the generation of strong fluorescence signals (Fig. 5A). Using this strategy, we successfully increased the signal size of the Arabidopsis centromere repeats on fixed nuclei (Fig. 5B). Compared with signals obtained by using streptavidin–FITC without TSA application the signal size increased roughly 2-fold in almost all nuclei. However, the centromere signals looked a bit fuzzier due to tyramide deposition near the target sequence (Fig. 5B). In summary, we successfully integrated TSA with CRISPR–FISH by utilizing biotinylated tracrRNA. This implementation has the potential to enhance the sensitivity of CRISPR–FISH.

## Discussion

CRISPR–FISH has demonstrated its ability to label repetitive DNA sequences in fixed samples (Ishii *et al.*, 2019; Potlapalli *et al.*, 2020). In this study, we have further refined this method to enhance its efficiency. This improvement aims to boost signal intensities, making it potentially feasible to label low-copy sequences in future. The evaluation of the ALFA-tag, designed to enhance DNA labeling, demonstrated the compatibility with the dCas9-based RNP complex, enabling precise DNA targeting without functional interference (Fig. 1B, C). In addition, the dCas9–ALFA-tag system efficiently labels repetitive sequences in both formaldehyde- and ethanol–acetic acid-fixed samples, highlighting its versatility. However, when examined under a fluorescence microscope, increasing ALFA-tag copies did not boost signal intensity when combined with a fluorescence-labelled NbALFA, possibly due to an allosteric hindrance in NbALFA–ALFA-tag binding, warranting further investigation (Matos, 2021).

The minibody, a fusion of an anti-ALFA single-domain antibody and a rabbit Fc domain, shows promise for signal amplification in our dCas9–ALFA system. While we observed no significant difference between single and triple ALFA-tagged dCas9 proteins on the N-terminus, dCas9 with more than three ALFA-tags revealed significant increases in the signal intensities. Considering dCas9 with 3×, 6× and 12× ALFA-tag copies, the increase in relative signal intensity is quite linear, starting with a mean of 3.47, going to 7.37 and 14.31 (Fig. 2E). The absence of any difference between constructs with one or three ALFA-tag copies suggests that not all of the three ALFA-tag can be recognized by minibodies, possibly due to limited space when ALFA-tags were densely placed. In contrast, constructs with higher copy numbers ALFA-tag provided sufficient space for an increased minibody binding. This finding is further supported by the fact that constructs where the ALFA-tag copies were distributed on both ends of the dCas9 (3 + 3× and 6 + 6×GS) showed significantly increased intensity levels when compared with constructs with identical copy numbers fused to only the N-terminus (6×, 6×GS, and 12×) (Fig. 3F). The dCas9 itself obviously acts as a spacer between the ALFA-tag copies allowing for a more efficient minibody binding. Only fusing 12× ALFA copies to both termini, creating a 24× version, did not further increase the signal. Instead additional background signals appeared, which made quantitative analysis impossible. This may be attributed to the diminished DNA labeling activity of the dCas9 due to the increased load of ALFA copies (12×) on either side.

Incorporating glycine-rich linkers between ALFA-tag copies did not significantly improve the signal intensity. Detection of Arabidopsis centromere repeats revealed that the 6× and 6×GS ALFA-tagged dCas9 proteins exhibited similar relative fluorescence intensities (mean: 7.37 versus 7.00), as illustrated in Fig. 3C. The 6 + 6×GS ALFA-tagged dCas9 protein revealed a mean of 16.29, which is the highest observed intensity of all constructs. However, based on our statistical analysis, the difference between 6 + 6×GS and 12× is caused by the spacing of the two 6× blocks and not by the GS linkers between the individual copies. It needs to be elucidated if incorporating longer linkers like XTEN (Schellenberger *et al.*, 2009; Guilinger *et al.*, 2014), providing more space for minibody binding to the ALFA-tag, might be an option to further increase the efficiency of the method in future.

When compared with standard FISH, CRISPR–FISH offers several advantages. It rapidly labels DNA sequences within minutes to hours, whereas standard FISH typically requires a longer timeframe, several hours up to days. CRISPR–FISH does not require a global denaturation and can be performed at different temperatures, ranging from 4 °C to 37 °C, potentially preserving the native cell and chromatin structure much better (Ishii *et al.*, 2019; Potlapalli *et al.*, 2020). Furthermore, CRISPR–FISH can easily be combined with immunostaining to co-image DNA sequences and chromatin-associated proteins (Ishii *et al.*, 2019). In contrast, FISH cannot be directly combined with immunostaining due to the denaturation process involved. Moreover, denaturation may potentially lead to the loss of DNA (Raap *et al.*, 1986) and chromatin-associated proteins (Mongelard *et al.*, 1999), impacting the results, especially when visualized at higher resolutions. Considering these advantages, the CRISPR–FISH method holds the potential for future development, particularly in combination with super-resolution imaging for studying the genome organization. Moreover, the ALFA-tag’s compatibility with glutaraldehyde (Götzke *et al.*, 2019), a superior fixative, might enhance our method’s capability to investigate DNA at an ultrastructural level using electron microscopy facilitated by nanobody-fused gold particles. Additionally, CRISPR RNP complexes could be directly delivered into plant protoplasts for live imaging in the future, a possibility that is not achievable with standard FISH techniques.

However, unlike standard CRISPR–FISH, a limitation of the developed CRISPR–FISH ALFA-tag system is its inability to simultaneously label different target sequences. This limitation could be addressed by using a second tag in parallel, such as the spot-tag (Braun *et al.*, 2016; Virant *et al.*, 2018), in conjunction with CRISPR–FISH. Furthermore, our study successfully achieved live imaging of telomeres in *N. benthamiana* nuclei through the fusion of six ALFA-tags to the C-terminal end of dCas9. Importantly, this method opens up the possibility of targeted pull-down experiments for DNA-binding proteins using an ALFA-tag-specific affinity resin (Kilisch *et al.*, 2021; Igreja *et al.*, 2022), offering valuable applications beyond telomere labeling.

The combination of TSA with indirect CRISPR–FISH resulted in strong labeling of Arabidopsis centromere sequences (Fig. 5B). Although the centromere signals were 2-fold larger in size, they appeared to be blurrier after TSA, possibly due to increased fluorescence molecule deposition near the target sequence. We suggest that using TSA’s sensitivity alongside the indirect CRISPR–FISH method could be an approach for visualizing small targets, like low-copy sequences that are otherwise too small to be detected, within structurally preserved chromatin in the future. However, for the integration of TSA with CRISPR–FISH, it is necessary to utilize a specialized TSA kit, potentially resulting in an increased overall cost of the assay. While TSA may introduce elevated background noise levels, this can be addressed by further optimizing conditions. Looking ahead, our study not only improves existing methods but also paves the way for future research. Application of CRISPR dCas9 holds the potential to become a valuable tool for labeling low-copy sequences within preserved chromatin and for executing precise *in vivo* pull-down assays targeting DNA-binding proteins. Furthermore, leveraging automation in microscopy and benefiting from enhanced sensitivity and rapid labeling capability, CRISPR–FISH shows potential for facilitating high-throughput analysis in both plant biology and diagnostic applications.

## Supplementary data

The following supplementary data are available at *JXB* online.

Fig. S1. Structure of expression vectors.

Fig. S2. CRISPR–ALFA-tag-based labeling of knob repeats, *Fok*I repeats and major satellite repeats on conventionally 3:1 (ethanol: acetic acid)-fixed chromosomes of *Z. mays*, *V. faba*, and mouse, respectively.

Fig. S3. Visualization of centromere repeats using dCas9 protein fused with multiples copies of ALFA-tag and NbALFA combination.

Fig. S4. Centromere labeling with dCas9 fused with 12 + 12× ALFA-tags.

Table S1. List of primer sequences used.

Table S2. List of synthetic ALFA-tag DNA fragment sequences, crRNAs, and oligo probes used.

Video S1. Live telomere imaging with dCas9–3×eGFP–ALFA-tag in *N. benthamiana* leaf nuclei over a 15 min period.

Video S2. Live telomere imaging with dCas9–3×eGFP–ALFA-tag in combination with mRuby–NbALFA in *N. benthamiana* leaf nuclei over a 15 min period. Panel from top to bottom: mRuby–NbALFA (red), dCas9–3×eGFP–ALFA-tag (green), and merged.

erae341_suppl_Supplementary_Figures_S1-S4

erae341_suppl_Supplementary_Tables_S1-S2

erae341_suppl_Supplementary_Video_S1

erae341_suppl_Supplementary_Video_S2

## Data Availability

All data supporting the findings of this study are available within the paper and within its supplementary materials published online. Methods are described in www.protocols.io, DOI: https://dx.doi.org/10.17504/protocols.io.n92ld85y7v5b/v1 (Potlapalli *et al*., 2024).
