# Supplementary material for: The potential of ALFA-tag and tyramide-based fluorescence signal amplification to expand the CRISPR-based DNA imaging toolkit
Source: J Exp Bot. 2024 Aug 6;75(20):6244–57. doi: 10.1093/jxb/erae341 (PMC11522987; doi:10.1093/jxb/erae341)
Supplement: erae341_suppl_Supplementary_Figures_S1-S4 [file erae341_suppl_supplementary_figures_s1-s4.pdf]

Fig. S1

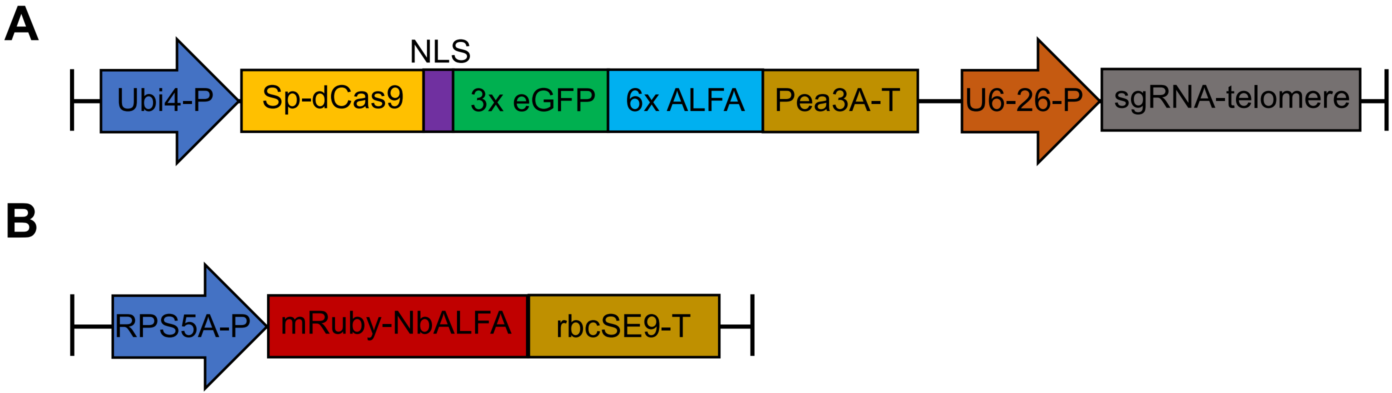

Fig. S1: Structure of expression vectors. (A) Illustration of the Sp. dCas9-3x eGFP-ALFA-tag construct. Transcription of sp-dCas9-3x eGFP-ALFA-tag was initiated by the parsley ubiquitin 4 promoter and terminated by the pea 3A terminator, respectively. The nuclear localization of dCas9 was achieved using an SV40 NLS DNA sequence Transcription of the sgRNA scaffold was initiated by the Arabidopsis ubiquitin 6 promoter. (B) Illustration of mRuby-NbALFA, where transcription was initiated by RPS5A promoter and terminated by the rbcSE9 terminator.

Fig. S2

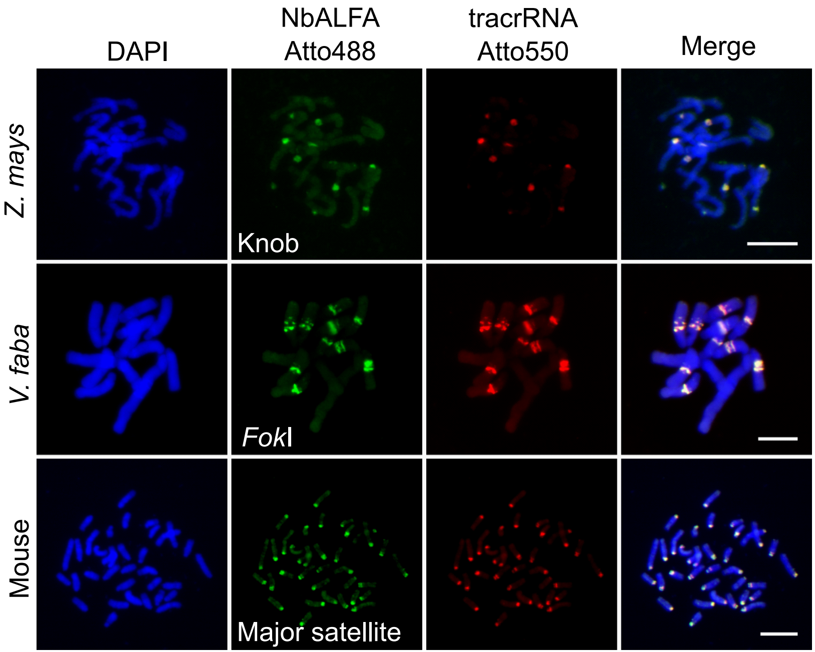

Fig. S2: CRISPR-ALFA-tag-based labeling of knob repeats, *Fok*I repeats and major satellite repeats on conventionally 3:1 (ethanol: acetic acid) fixed chromosomes of *Z. mays*, *V. faba* and mouse, respectively. Green signals represent NbALFA Atto488, and red signals correspond to ATTO550-tagged tracrRNA. Chromosomes were counterstained with DAPI (in blue). Scale bars: 10 µm.

Fig. S3

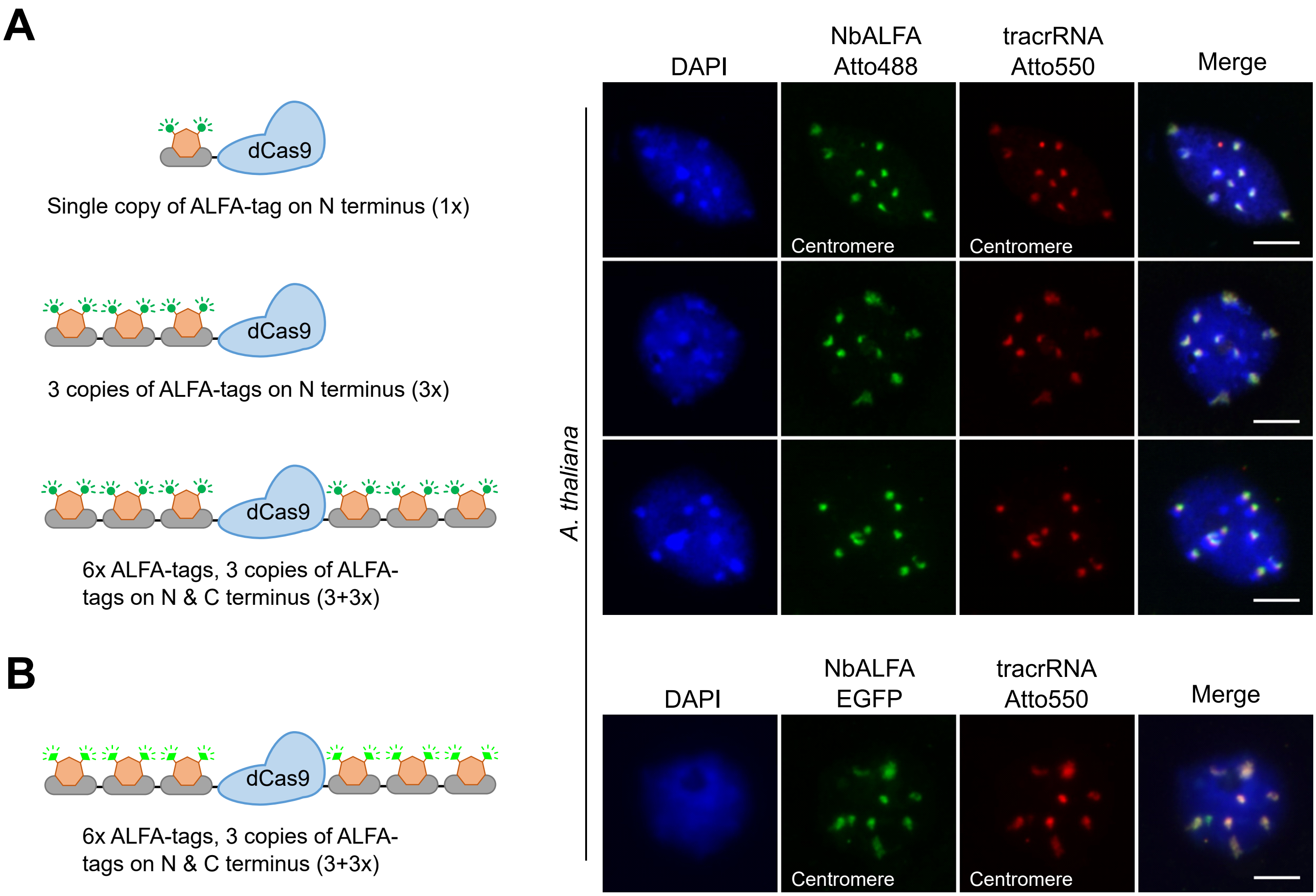

Fig. S3: Visualization of centromere repeats using dCas9 protein fused with multiple copies of ALFA-tag and NbALFA combination. (A) Sketch illustrating the dCas9 protein fused with either (upper)1x or (middle) 3x ALFA copies at the N-terminus and (lower) 6x copies (3x on each end) of dCas9 protein. On the right side, visualization of centromere repeats using dCas9-ALFA-tag proteins and detected with NbALFA conjugated to ATTO488. (B) Sketch illustrating the dCas9 protein fused with 6x copies (3x on each end) of ALFA-tags. On the right side, the labeling of A. thaliana centromeres with dCas9-ALFA-tag along with EGFP-NbALFA. Green signals represent (A) NbALFA Atto488 or (B) EGFP NbALFA, and red signals correspond to ATTO550-tagged tracrRNA. Nuclei were counterstained with DAPI (in blue). Scale bars: 10 µm.

Fig. S4

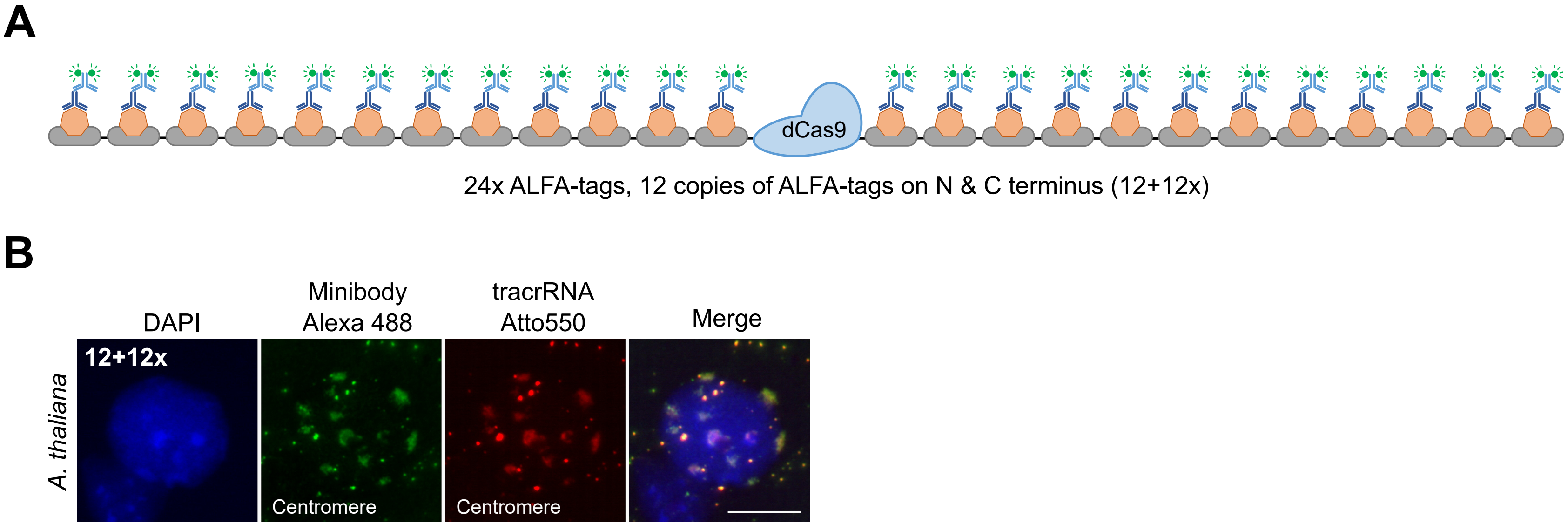

Fig. S4: Centromere labeling with dCas9 fused with 12+12x ALFA-tags: (A) Sketch illustrating the dCas9 protein fused with 24x copies (12x on each end) of ALFA-tags. (B) Visualization of centromere repeats using dCas9 protein fused with 12+12x ALFA copies. Detection was performed using minibody and anti-rabbit Alexa 488. dCas9 fused with 12+12x ALFA exhibited minimal labeling. Green signals represent anti-rabbit Alexa 488, and red signals correspond to ATTO550-tagged tracrRNA. Nuclei were counterstained with DAPI (in blue). Scale bars: 10 µm.
