# Supplementary material for: The potential of ALFA-tag and tyramide-based fluorescence signal amplification to expand the CRISPR-based DNA imaging toolkit
Source: J Exp Bot. 2024 Aug 6;75(20):6244–57. doi: 10.1093/jxb/erae341 (PMC11522987; doi:10.1093/jxb/erae341)
Supplement: erae341_suppl_Supplementary_Tables_S1-S2 [file erae341_suppl_supplementary_tables_s1-s2.pdf]

Table. S1: List of primer sequences used.

Name of primer	Sequence (5`-3`)	Purpose
dCas9-NcoI-F	CATGCCATGGATAAGAAGTACTCTATCGGACTC	F and R primers are required to amplify the Sp.dCas9 sequence from dCas9:3xPP7: GFP for cloning onto the pET22b+ vector
dCas9-HindIII-F	CCCAAGCTTAACCTTCCTCTTCTTCTTAGGATCAG	
1x ALFA-N ter-F	CCAGCCGGCGATGGCAATGCCATCACGTTTGGAAGAGGAACTGAGACGCCGCTTAACTGAACCT	F and R primers are required to amplify a single copy of the ALFA tag from the ALFA synthetic fragment for cloning onto the N-terminus of the pET22b+-dCas9 vector
1x ALFA-N ter-R	GTCCGATAGAGTACTTCTTATCAGGTTCAGTTAAGCGGCGTCTCAGTTCCTCTTCCAAACGTGATGG	
1x ALFA-C ter-F	AAGCTTGGCTCTCCATCACGTTTGGAAGAGGAACTGAGACGCCGCTTAACTGAACCT	F and R primers are required to amplify a single copy of the ALFA tag from the ALFA synthetic fragment for cloning onto the C-terminus of the pET22b+-dCas9 vector
1x ALFA-C ter-R	GGGCCGCAGGTTCAGTTAAGCGGCGTCTCAGTTCCTCTTCCAAACGTGATGG	
3x ALFA-N ter-F	CATGCCATGGCCCCCTCACGATTAGAAGAGGAACT	F and R primers are required to amplify three copies of the ALFA tags from the 3x ALFA synthetic fragment for cloning onto the N-terminus of the pET22b+-dCas9 vector
3x ALFA-N ter-R	CATGCCATGGGCGGCTCGGTCAAACGACG	
3x ALFA-C ter-F	AAGCTTCCCTCACGATTAGAAGAGGAACT	F and R primers are required to amplify three copies of the ALFA tags from the 3x ALFA synthetic fragment for cloning onto the C-terminus of the pET22b+-dCas9-3xALFA tag (N-ter) vector (3+3x)
3x ALFA-C ter-R	GCGGCCGCCGGCTCGGTCAAACGACG	
6x ALFA-N ter-F	CATGCCATGGCCCCCTCACGATTAGAAGAGGAACT	F and R primers are required to amplify six copies of the ALFA tags from the 6xALFA synthetic fragment for cloning onto the N-terminus of the pET22b+-dCas9 vector
6x ALFA-N ter-R	CATGCCATGGGAGGTTCGGTCAGACGCCT	
12x ALFA-N ter-F	CATGCCATGGCCCCCTCACGATTAGAAGAGGAACT	F and R primers are required to amplify twelve copies of the ALFA tags from the 12xALFA synthetic fragment for cloning onto the N-terminus of the pET22b+-dCas9 vector
12x ALFA-N ter-R	CATGCCATGGCCGGCGGCTCGGTTAAACGCCTAC	
12x ALFA-C ter-F	CCCAAGCTTCCCTCACGATTAGAAGAGGAACT	F and R primers are required to amplify twelve copies of the

12x ALFA-C ter-R	AAATAGCGGCCGCGGACGGCTCGGTTAAACGCCTAC	ALFA tags from the 12xALFA synthetic fragment for cloning onto the N-terminus of the<br><br>pET22b+-dCas9-12xALFA tag (N-ter) vector (12+12x)
6x GS ALFA-N ter-F	CATGCCATGGccGGTGGCGGAGGGTCTCC	F and R primers are required to amplify six copies of the ALFA tags from the 6xALFA (GGGGS linker) synthetic fragment for cloning onto the N-terminus of the pET22b+-dCas9 vector
6x GS ALFA-N ter-R	CATGCCATGGGAGGTTCGGTCAGACGCCT	
6x GS ALFA-C ter-F	CCCAAAGCTTGGTGGCGGAGGGTCTCC	F and R primers are required to amplify six copies of the ALFA tags from the 6xALFA (GGGGS linker) synthetic fragment for cloning onto the N-terminus of the pET22b+-dCas9-6xALFA GS (N-ter) vector
6x GS ALFA-C ter-R	AAATAGCGGCCGCGGAAGGTTCGGTCAGACGCCT	
NbALFA-F	TTGAAGACAAAGGTGAAGTGCAGCTTCAGGAGAGTG	F and R primers are required to amplify NbALFA from pET51b(+)_eGFP NbALFA, (Addgene 136626) for cloning onto pAGM1299
NbALFA-R	TTGAAGACAACGAATTATGACGACACAGTGACCTG	
mRuby-F	TTGAAGACAAAGGTGGCTCTGGATCGGGGTCGGGCTCAATGGTGTCTAAGGGCGAAGAGCTG	F and R primers are required to amplify mRuby from Sp-dCas9-mRuby (DREISSIG et al. 2017), for cloning onto pICH41258
mRuby-R	TTGAAGACTTCGAAAAGCTTACTTGTACAGCTCGTCCATCCCACC	
RPS5A Pro-F	TTGAAGACAAGGAGCTCAACTTTTGATTCGC	F and R primers are required to amplify RPS5A promoter for cloning onto pICH41295
RPS5A Pro-R	TTGAAGACAACATTGCTGTGGTGAGAGAAACAGAGC	
rbcSE9 ter-F	TTGAAGACATCTCATTCGTAGTAATTATGGCATTGGGAAAACTG	F and R primers are required to amplify rbcSE9 terminator for cloning onto pICH9121
rbcSE9 ter-R	TTGAAGACAACTCGAGCGTGTTTTACTCCTCATATTAACTTCGGTC	
Pea3A ter-F	TTGAAGACAAGCTTCAGGCCTCCCAGCTTTCGT	F and R primers are required to amplify Pea3A promoter from Sp-dCas9-eGFP (DREISSIG et al. 2017), for cloning onto pICH41276
Pea3A ter-R	TTGAAGACAAAGCGAAGCCTATACTGTACTTAACTTGATTGCATAATTACTTGA	
3x eGFP-F	TTGAAGACAAAGGTGGCTCTGGATCGGGGTCG	F and R primers are required to amplify 3x eGFP from Sp-

3x eGFP-R	TTGAAGACTTCGAATTCAGGCGTAGCGCTCTCG	dCas9-eGFP (DREISSIG et al. 2017), for cloning onto pAGM1299
Sp.dCas9-F	TTGAAGACAAAATGGATAAGAAGTACTCTATCGGACTCGC	F and R primers are required to amplify Sp.dCas9 from Sp-dCas9-eGFP (DREISSIG et al. 2017), for cloning onto pICH41258
Sp.dCas9-R	TTGAAGACATACCTTTTGAGCCCGACCCCGAT	
Ubi4 pro-F	TTGAAGACAAGGAGAAAAATTACGGATATGAATATAGGCATATCCG	F and R primers are required to amplify Ubi4 promoter from Sp-dCas9-eGFP (DREISSIG et al. 2017), for cloning onto pICH41295
Ubi4 pro-R	TTGAAGACAACATTGCTGCACATACATAACATATCAAGATCAG	
6x ALFA (GS)-F	TTGAAGACAATTCGGTAGCATCGATGGTAGCCCATC	F and R primers are required to amplify 6x ALFA GS (linker) from pET22b+-dCas9-6xALFA GS, for cloning onto pAGM1301
6x ALFA (GS)-R	TTGAAGACAAAAGCTTATTCGGTCAGACGCCTACGG	
Telomere sgRNA-F	TGTGGTCTCAATTGGGGTTTAGGGTTTAGGGTTTGTTTTAGAGCTAGAAATAGCAAG	F and R primers are required to amplify Arabidopsis Telomere sgRNA from dCas9:3xPP7: GFP, for cloning onto pICH47742 along with pICH41295-U6 (At-26) promoter to make level 1 vector
sgRNA scaffold-R	TGTGGTCTCAAGCGTAATGCCAACTTTGTAC	
At-U6(26)-Pro-F	TGTGAAGACAAGGAGCTTTTTTTCTTCTTCTTCGTTCATACAGTTTTTTTTTGTTTAT	F and R primers are required to amplify U6 (At-26) promoter from dCas9:3xPP7: GFP, for cloning onto pICH41295
U6(At-26)-Pro-R	TTGAAGACAACATTCACTACTTCGACTCTAGCTGTATATAAACTCAG	

Table. S2: List of synthetic ALFA-tag DNA fragment sequences, crRNAs and oligo probes used.

ALFA tag DNA fragment sequences	1xALFA tag sequence	CCATCACGTTTGGAAGAGGAACTGAGACGCCGCTTAACTGAACCT
	3x ALFA<br><br>tags sequence	CCCTCACGATTAGAAGAGGAACTAAGGAGACGCTTAACGGAACCGC<br>CGAGCCGTCTCGAAGAAGAGCTCAGGAGACGCCTGACAGAACCAC<br>CGTCCAGATTGGAAGAGGAGCTGCGTCGTCGTTTGACCGAGCCG
	6x ALFA tags sequence	CCCTCACGATTAGAAGAGGAACTAAGGAGACGCTTAACGGAACCGC<br>CGAGCCGTCTCGAAGAAGAGCTCAGGAGACGCCTGACAGAACCAC<br>CGTCCAGATTGGAAGAGGAGCTGCGTCGTCGTTTGACCGAGCCGC<br>CATCTCGCTTAGAGGAGGAACTGCGCAGGAGACTCACTGAACCGCC<br>GTCAAGACTCGAGGAAGAACTCCGCAGAAGGTTAACTGAGCCACCC<br>AGCAGATTAGAGGAGGAACTCCGTAGGCGTCTGACCGAACCT
	12x ALFA tags sequence	CCCTCACGATTAGAAGAGGAACTAAGGAGACGCTTAACGGAACCGC<br>CGAGCCGTCTCGAAGAAGAGCTCAGGAGACGCCTGACAGAACCAC<br>CGTCCAGATTGGAAGAGGAGCTGCGTCGTCGTTTGACCGAGCCGC<br>CATCTCGCTTAGAGGAGGAACTGCGCAGGAGACTCACTGAACCGCC<br>GTCAAGACTCGAGGAAGAACTCCGCAGAAGGTTAACTGAGCCACCC<br>AGCAGATTAGAGGAGGAACTCCGTAGGCGTCTGACCGAACCTCCGT<br>CTAGACTCGAAGAGGAGCTAAGGCGCAGGCTGACGGAGCCCCCTT<br>CACGTTTGGAGGAAGAGTTACGTAGGCGCTTGACTGAACCCCCGTC<br>TCGTTTAGAAGAAGAGCTACGTAGAAGATTGACGGAGCCGCCCTCT<br>CGTCTTGAGGAAGAACTTAGGCGTAGATTAACCGAACCACCATCCC<br>GTCTGGAAGAAGAGTTAAGGCGTAGATTGACTGAGCCACCCAGCCG<br>ACTCGAGGAAGAGCTTCGTAGGCGTTTAACCGAGCCG
	6x ALFA tags sequence separated by GGGGS linkers	GGTGGCGGAGGGTCTCCCTCACGATTAGAAGAGGAACTAAGGAGA<br>CGCTTAACGGAACCGGGCGGTGGAGGTTCCCCGAGCCGTCTCGAA<br>GAAGAGCTCAGGAGACGCCTGACAGAACCAGGAGGTGGCGGGTCA<br>CCGTCCAGATTGGAAGAGGAGCTGCGTCGTCGTTTGACCGAGCCG<br>GGGGGTGGCGGATCGCCATCTCGCTTAGAGGAGGAACTGCGCAGG<br>AGACTCACTGAACCGGGGGGAGGCGGTAGTCCGTCAAGACTCGAG<br>GAAGAACTCCGCAGAAGGTTAACTGAGCCAGGGGGAGGCGGTAGT<br>CCCAGCAGATTAGAGGAGGAACTCCGTAGGCGTCTGACCGAACCT
crRNA sequences	*A. thaliana* centromere	5`- TTGAGAAGCAAGAAGAAGGT -3`
	Z. mays<br><br>knob	5`- AAGGAAACATATGTGGGGTG -3`
	V. Faba<br><br>*Fok*I	5`- CGAGATTTTTGTTACTCCAA -3`
	Mouse<br><br>MS	5`- CAGTTTTCTCGCCATATTCC -3`

Oligo probe	*A. thaliana* telomere	5`Cy3 –GGGTTTAGGGTTTAGGGTTT-3`
